# Supplementary material for: Size Scaling of the Electrochemical Performance of Ti3C2Tx MXene Microelectrode Arrays for Electrophysiological Recording and Stimulation
Source: bioRxiv. 2026 Mar 23:2026.03.19.712102. Preprint. [Version 1] doi: 10.64898/2026.03.19.712102 (PMC13041874; doi:10.64898/2026.03.19.712102)
Supplement: Supplement 1 [file media-1.pdf]

Supporting Information

Size Scaling of the Electrochemical Performance of $Ti_3C_2T_x$ MXene Microelectrode Arrays for Electrophysiological Recording and Stimulation

*Spencer R. Averbeck[1,2], Raghav Garg[2,3], Royce Dong[1,2], Daryl Hurwitz[2], Nicholas V. Apollo[4], Michael S. Beauchamp[4], Flavia Vitale[1,2,3,5,6]**

[1] S. R. Averbeck, R. Dong, F. Vitale
Department of Bioengineering,
University of Pennsylvania,
Philadelphia, Pennsylvania – 19104, USA.
E-mail: vitalef@pennmedicine.upenn.edu

[2] S. R. Averbeck, R. Garg, R. Dong, D. Hurwitz, N.V. Apollo, F. Vitale
Center for Neuroengineering and Therapeutics,
University of Pennsylvania,
Philadelphia, Pennsylvania – 19104, USA.

[3] R. Garg, F. Vitale
Department of Neurology,
University of Pennsylvania,
Philadelphia, Pennsylvania – 19104, USA.

[4] M. S. Beauchamp
Department of Neurosurgery,
University of Pennsylvania,
Philadelphia, Pennsylvania – 19104, USA.

[5] F. Vitale
Department of Physical Medicine and Rehabilitation,
University of Pennsylvania,
Philadelphia, Pennsylvania – 19104, USA.

[6] F. Vitale
Center for Neurotrauma, Neurodegeneration, and Restoration,
Corporal Michael J. Crescenz Veterans Affairs Medical Center,
Philadelphia, Pennsylvania – 19104, USA.

SUPPLEMENTARY FIGURES

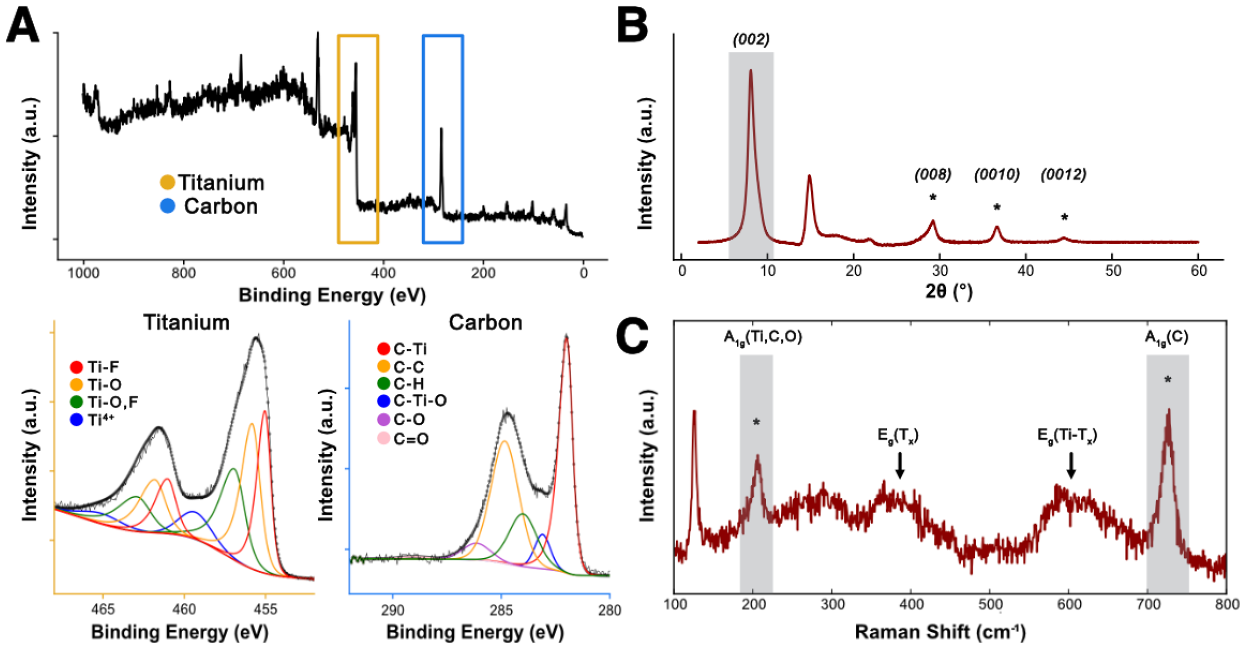

Figure S1. Structural analysis of MILD-synthesized $Ti_3C_2T_x$ MXene. (A) *Top:* XPS Survey Spectra of $Ti_3C_2T_x$ MXene, highlighting Titanium and Carbon peaks. *Bottom Left:* Core-level Ti *2p* spectra of $Ti_3C_2T_x$ MXene. *Bottom Right:* Core-level C *1s* spectra of $Ti_3C_2T_x$ MXene **(B)** XRD spectra of $Ti_3C_2T_x$ MXene, highlighting characteristic 002 peak. **(C)** Raman spectra of $Ti_3C_2T_x$ MXene, highlighting characteristic out-of-plane A_{1g} peaks as well as the in-plane E_g peaks.

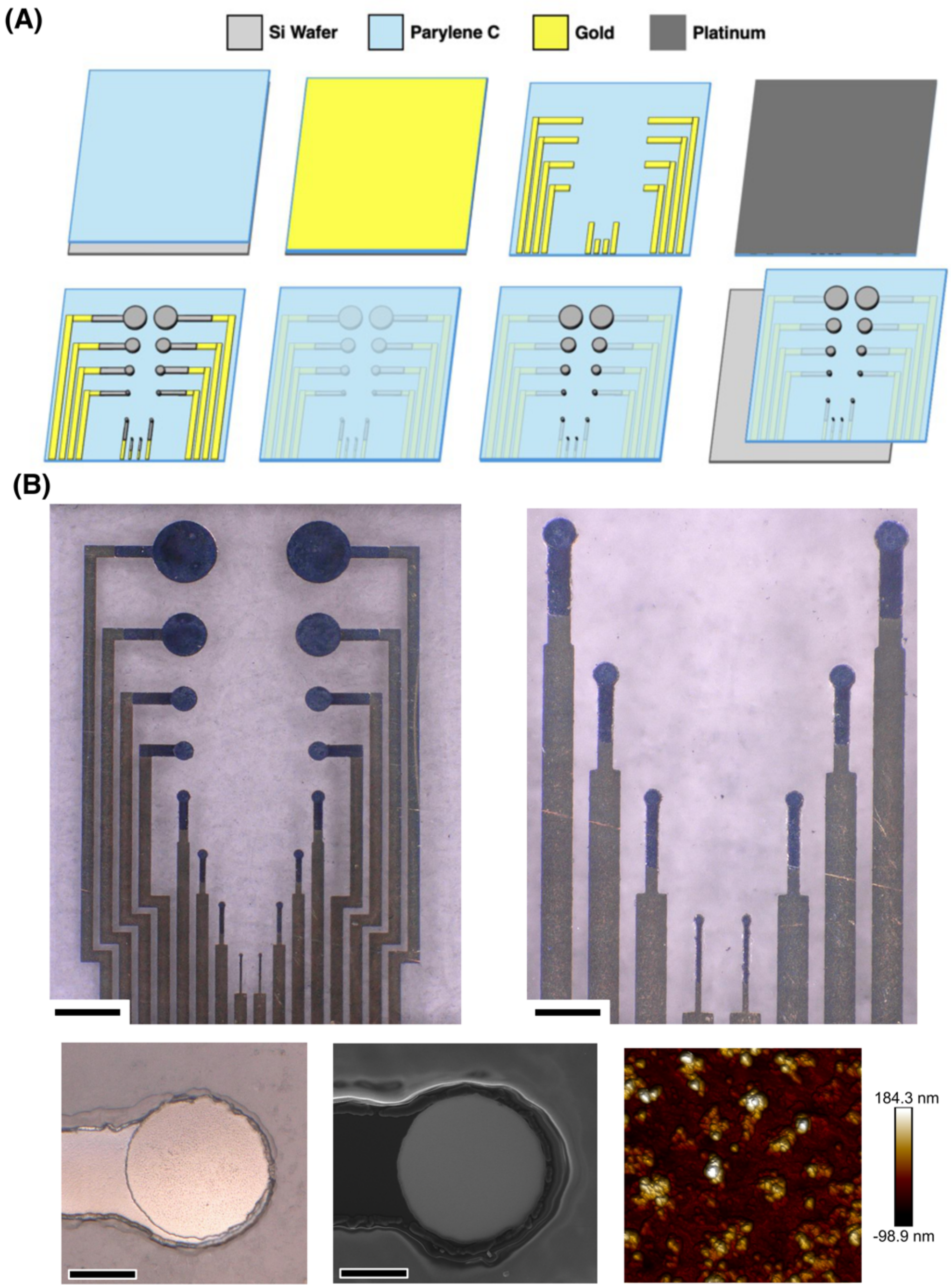

Figure S2. Fabrication schematic and images of Pt microelectrodes. (A) Schematic of the fabrication flow. **(B)** *Top:* Optical image of Pt microelectrodes (scale bar 500 µm) with zoomed-in insets of 100 µm, 75 µm, 50 µm, and 25 µm contacts. Scale bar 150 µm. *Bottom.* From left to right: optical, SEM, and AFM images of 100 µm Pt contacts. Scale bar 30 µm.

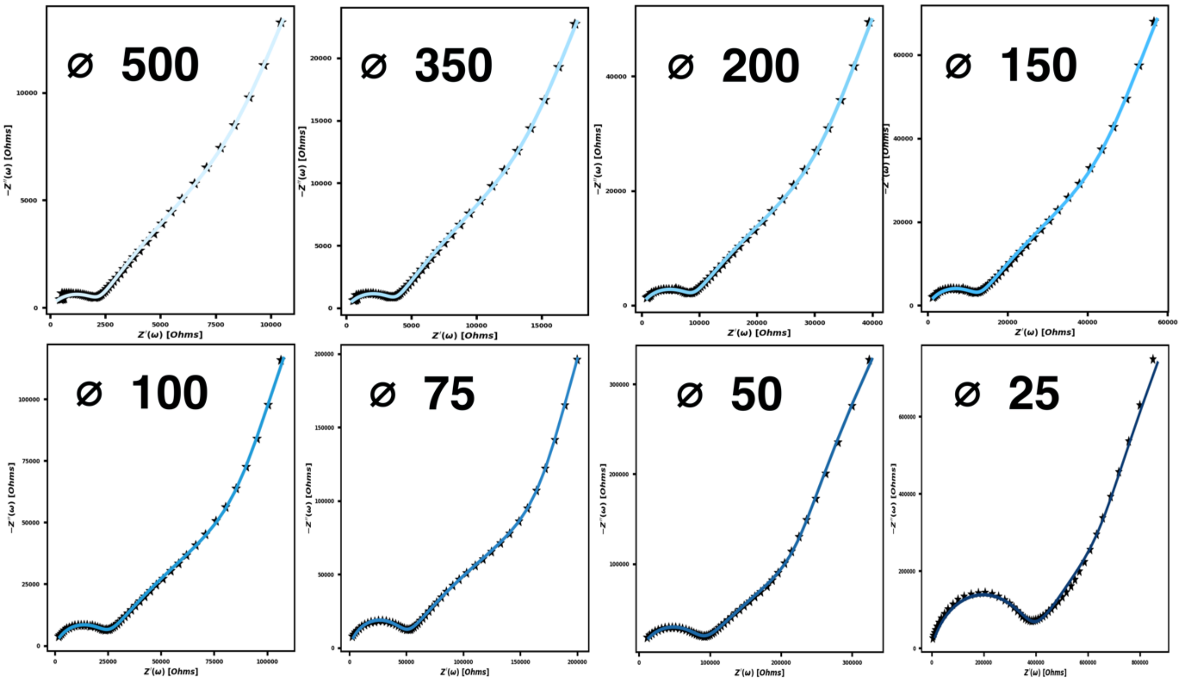

Figure S3. $Ti_3C_2T_x$ microelectrodes: Nyquist plots and equivalent circuit model fittings for all contact diameters.

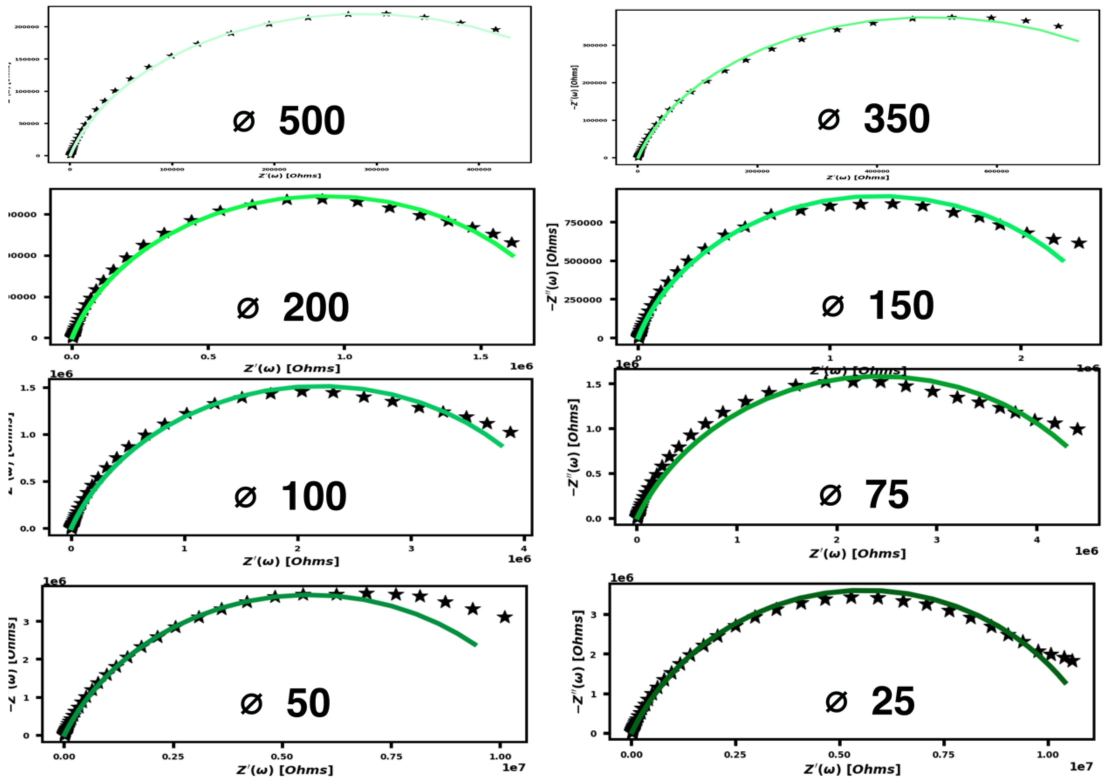

Figure S4. Pt microelectrodes: Nyquist plots and equivalent circuit model fittings for all contact diameters.

A

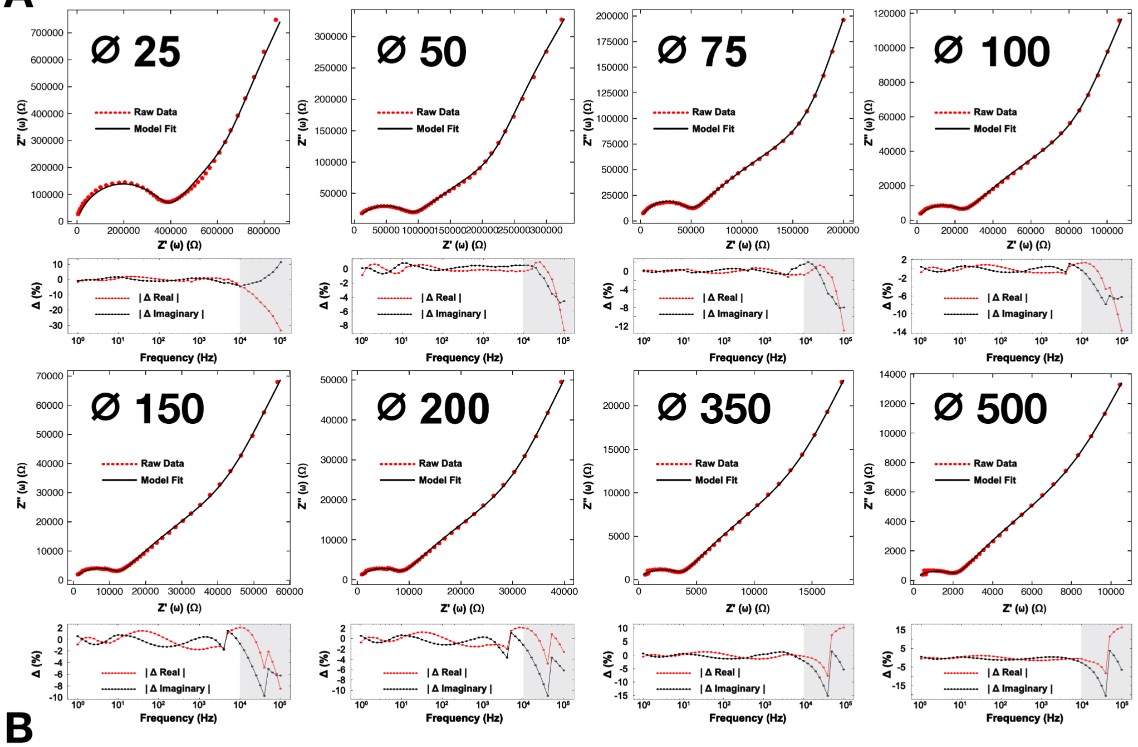

B

Electrode Diameter	25 μm	50 μm	75 μm	100 μm	150 μm	200 μm	350 μm	500 μm
RMS $Error_{Real}$ (%)	1.24	0.38	0.45	0.68	1.14	1.11	0.77	0.82
RMS % $Error_{Imaginary}$ (%)	1.39	0.41	0.47	0.51	0.77	1.00	0.85	0.80

Figure S5. Error Analysis and Model Fit Residuals. **(A)** For each contact diameter, *(Top)* Raw and fitted Nyquist plot, (*Bottom)* Real and imaginary model fit residuals across all frequencies, highlighting low error for all frequencies that satisfy KK-linearity consistency checks (1 Hz – 10 kHz). Grey-highlighted regions in residuals represent content that did not meet a KK consistency check within 2.5%. **(B)** RMS error for real and imaginary components of residuals within the KK-linear region, expressed as RMS percent error, where residuals at each frequency are normalized by the magnitude of the measured impedance $|Z_{meas}|$ for each electrode diameter.

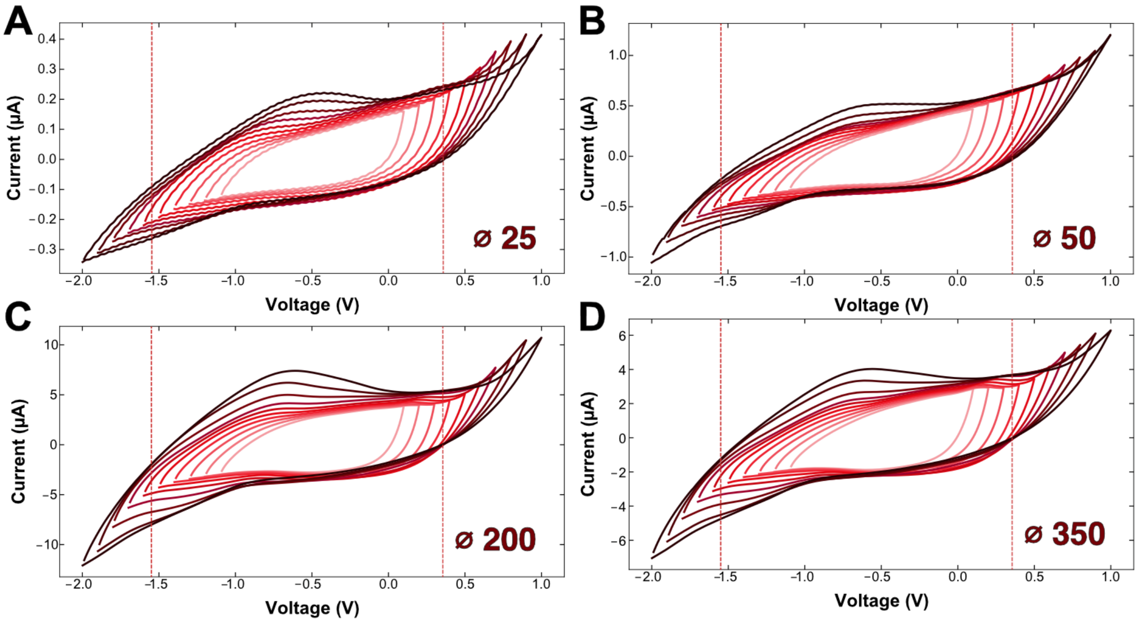

Figure S6. Water window determination for $Ti_3C_2T_x$ MXene thin-film microelectrodes. (A-D) Expanding potential windows delineating anodic and cathodic voltage limits for $Ti_3C_2T_x$ MXene thin-film microelectrodes at diameters of **(A)** 25 μm, **(B)** 50 μm, **(C)** 200 μm, and **(D)** 350 μm.

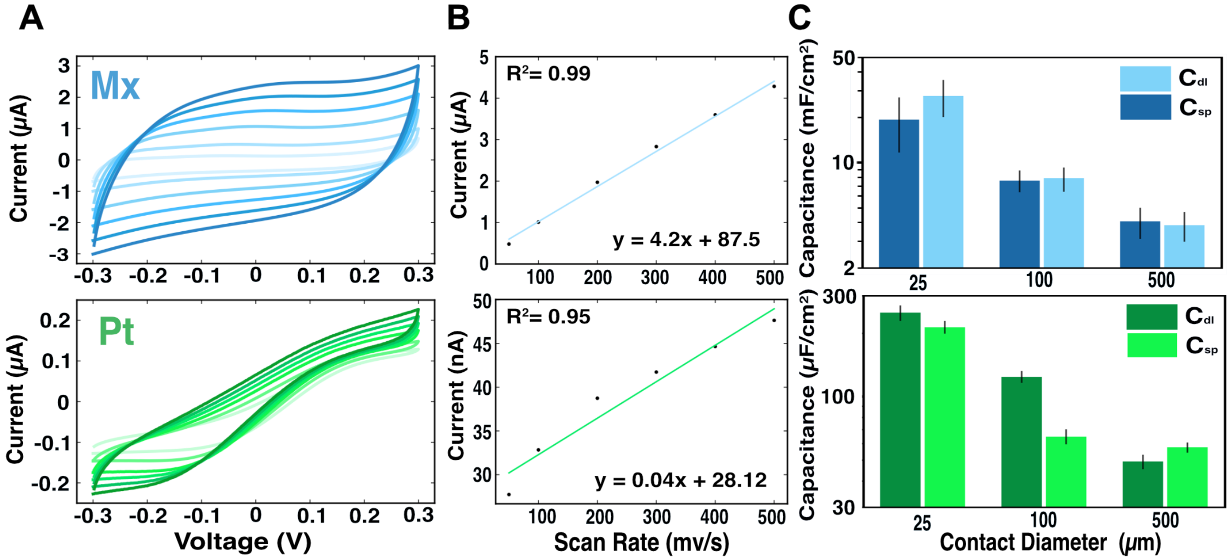

Figure S7. Specific capacitance from CVs vs C_{dl} from the equivalent circuit model. (A) Cyclic voltammograms at varying scan rates (50 mV s^{-1}, 100 mV s^{-1}, 200 mV s^{-1}, 300 mV s^{-1}, 400 mV s^{-1}, 500 mV s^{-1}) for 350 μm microelectrodes. *Top:* $Ti_3C_2T_x$, *Bottom:* Pt. **(B)** Current vs. scan rate for *Top:* $Ti_3C_2T_x$ and *Bottom:* Pt microelectrodes. **(C)** Comparison of the capacitance calculated from **(B)** and from the equivalent circuit model for *Top:* $Ti_3C_2T_x$, *Bottom:* Pt microelectrodes.

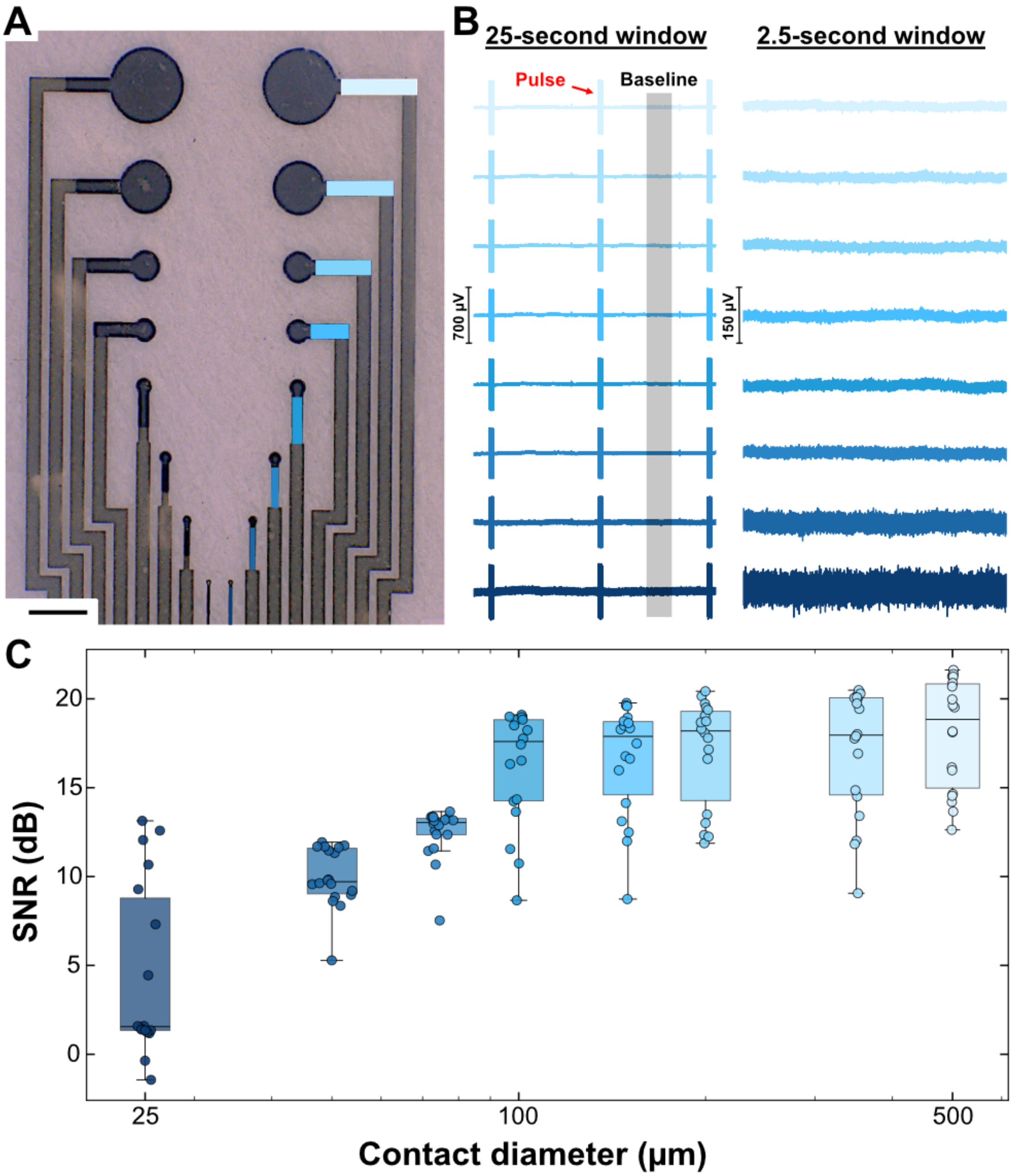

Figure S8. Relating electrode size to SNR. (A) Representative image of the $Ti_3C_2T_x$ microelectrode array. Scale bar = 500 µm. **(B)** *Left:* Three representative stimulation pulses from each electrode over a 25-second window, with the color corresponding to the electrode, on the left. *Right:* Zoomed-in 2.5-second clip of noise floor for each electrode diameter. **(C)** SNR as a function of contact diameter. N = 3 devices, 10 stim pulses per recording.

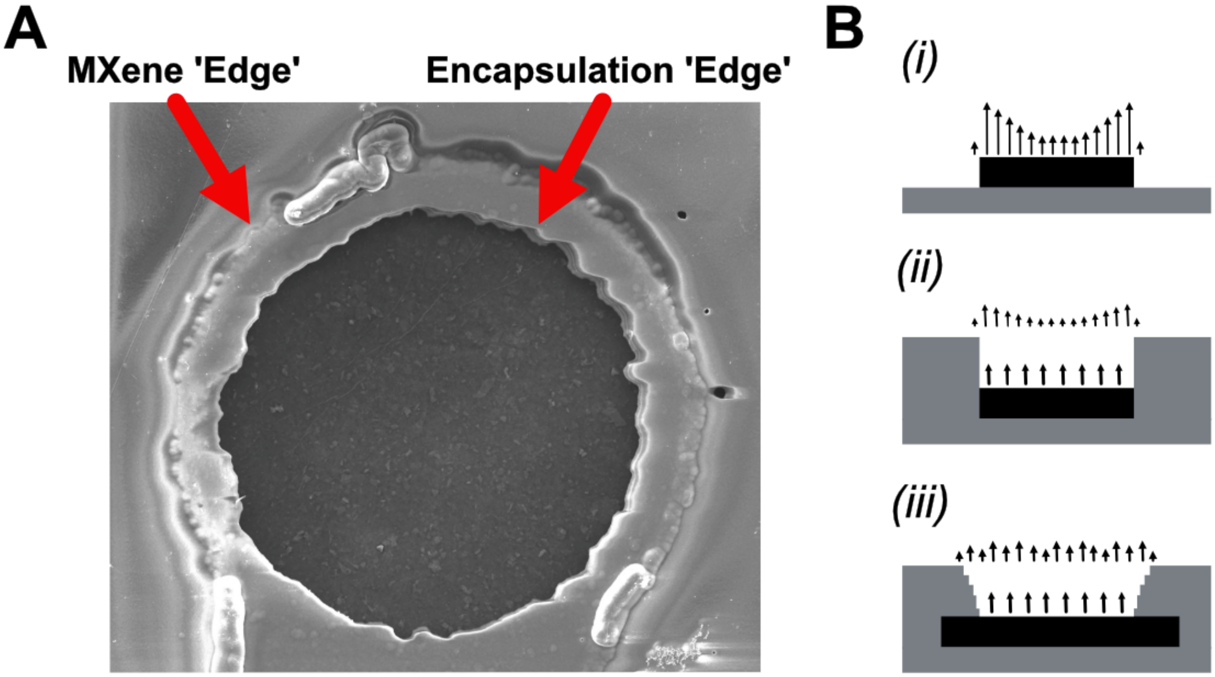

Figure S9. $Ti_3C_2T_x$ MXene thin-film microelectrode 'edge effects' considerations. (A) SEM image of a 100 μm diameter electrode contact. Red arrows point to the 'edge' of the spray-coated MXene, defined by the photoresist during fabrication, and the encapsulation edge, defined by the RIE etching of the parylene C encapsulation. **(B)** Expected charge distribution of electrodes fabricated with a *(i)* surface-mounted, *(ii)* simple recessed, and *(iii)* conically / irregularly recessed electrode.[1]

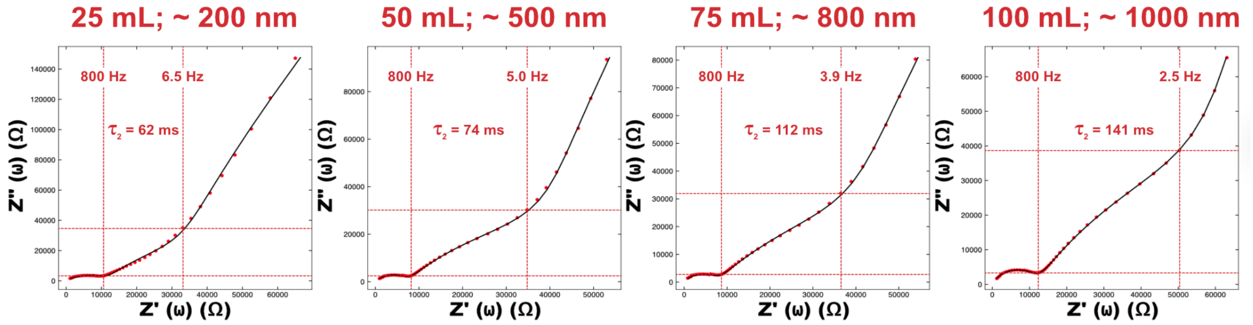

Figure S10. Emergence of low-frequency RC feature with increasing film thickness. As the thickness of the spray-coated $Ti_3C_2T_x$ MXene film increases for identically fabricated, 200 μm diameter electrodes, the frequency width and characteristic prominence of the 'bulk-layered' RC time-constant grow. N= 8 electrode samples per film thickness.

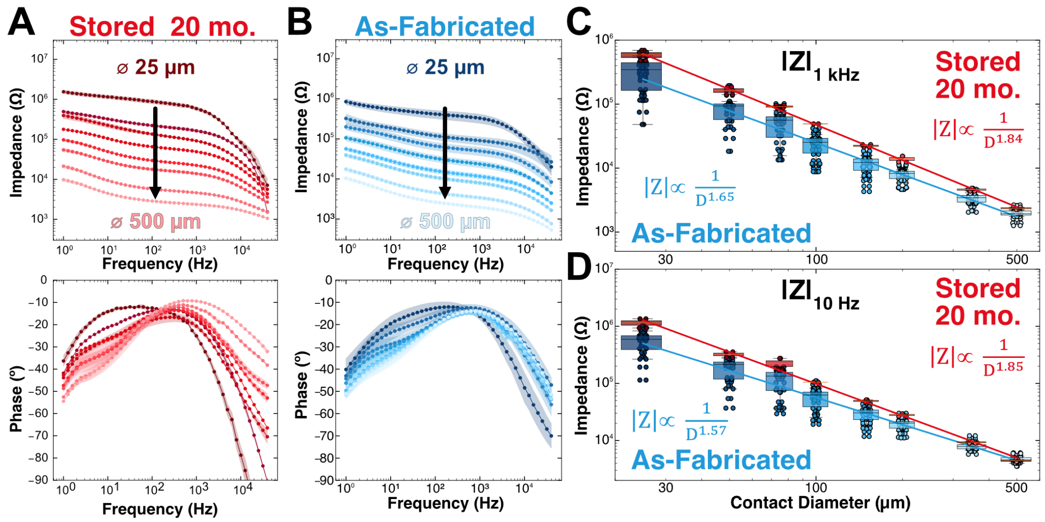

Figure S11. Shelf-life of $Ti_3C_2T_x$ MXene microelectrodes after 20 months: electrochemical impedance spectroscopy. Impedance modulus and phase spectra of $Ti_3C_2T_x$ microelectrodes of varying diameter (**A**) stored for 20 months, and (**B**) as-fabricated. Individual points represent the mean, shaded areas represent ± 1 standard deviation. (**C, D**) Impedance modulus at (**C**) 1 kHz and (**D**) 10 Hz as a function of electrode diameter.

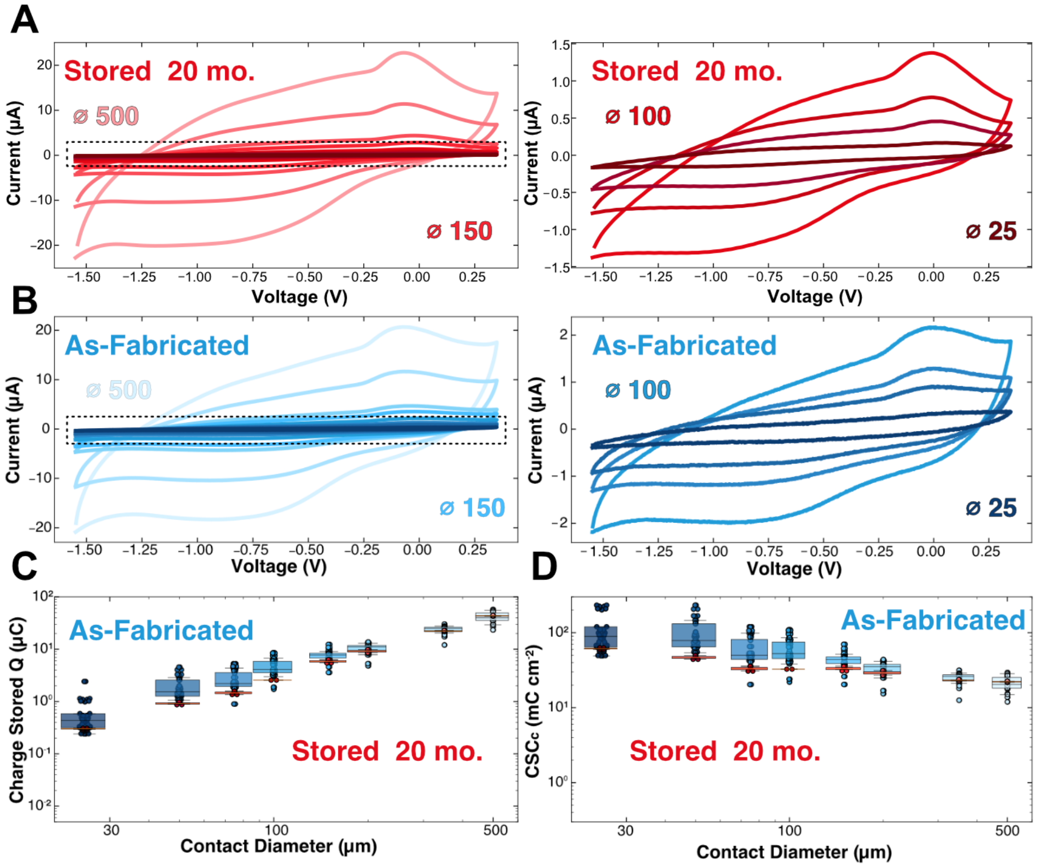

Figure S12. Shelf-life of $Ti_3C_2T_x$ MXene microelectrodes after 20 months: cyclic voltammetry and charge storage capacity. (A) *Left:* Cyclic voltammograms averaged across electrodes (n_{stored} = 4 electrodes, each size) of $Ti_3C_2T_x$ MXene microelectrodes stored for over 20 months from -1.55 V (cathodic limit) to 0.35 V (anodic limit). *Right:* Zoomed-in voltammograms

for contacts ranging from 100 µm to 25 µm. **(B)** *Left:* Cyclic voltammograms of freshly fabricated $Ti_3C_2T_x$ MXene microelectrodes averaged across electrodes ($n_{as\text{-}fabricated}$ = 34 electrodes, each size) from -1.55 V (cathodic limit) to 0.35 V (anodic limit). *Right:* Zoomed-in voltammograms for contacts ranging from 100 µm to 25 µm. **(C)** Charge stored across all contact sizes for aged and as-fabricated $Ti_3C_2T_x$ devices. **(D)** Cathodic charge storage capacity across all contact sizes of aged and freshly fabricated $Ti_3C_2T_x$ devices.

SUPPLEMENTARY TABLES

Table S1. Impedance values at 1 kHz and 10 Hz for $Ti_3C_2T_x$ and Pt devices.

Contact Diameter	Frequency (Hz)	$Ti_3C_2T_x$		Platinum	
		Mean (kΩ)	S.D. (kΩ)	Mean (kΩ)	S.D. (kΩ)
500 µm	1,000	1.97	0.34	1.11	0.11
	10	4.67	0.58	74.4	12.2
350 µm	1,000	3.40	0.67	1.92	0.20
	10	8.07	1.41	145	19.6
200 µm	1,000	7.97	1.80	4.32	1.01
	10	21.2	5.72	347	201
150 µm	1,000	11.6	3.57	6.70	1.77
	10	28.3	7.98	529	277
100 µm	1,000	23.6	8.97	13.7	4.22
	10	58.2	22.7	1,100	527
75 µm	1,000	49.8	23.6	18.5	7.13
	10	117	50.8	1,390	755
50 µm	1,000	90.8	42.0	49.0	17.6
	10	188	71.9	2,670	1,170
25 µm	1,000	328	168	140	74.7
	10	550	218	4,370	1,960

Table S2. Summary of all equivalent circuit model parameters for 200 µm $Ti_3C_2T_x$ and Pt microelectrodes.

Electrode	Circuit Elements	Values from Fitting	Calculated Cdl ($\mu F/cm^2$)
**$Ti_3C_2T_x$**	R_s (kΩ)	**0.880 ± 0.211**	
	α_0	.680 ± .014	**57.3 ± 4.31**
	Y_0 (S s^α)	(6.78 ± .061) x10^{-8}	
	R_{ct} (kΩ)	**9.15 ± 0.997**	
	R_{eq} (kΩ)	102 ± 13.7	
	W_{diff0} (kΩ)	0.276 ± .022	
	W_{diff1} (s)	(2.58 ± .103) x10^{-4}	
	α_1	.640 ± .019	**5,960 ± 982**
	Y_1 (S s^α)	(3.63 ± .161) x10^{-6}	
**Platinum**	R_s (kΩ)	**0.994 ± 0.153**	
	α_0	.814 ± .023	**66.9 ± 4.87**
	Y_0 (S s^α)	(3.39 ± .049) x10^{-8}	
	R_{ct} (kΩ)	**1,850 ± 236**	

Table S3. Summary of key model parameters for $Ti_3C_2T_x$ and Pt devices from EIS fitting.

Contact Diameter	Frequency (Hz)	$Ti_3C_2T_x$		Platinum		Theory
		Mean (kΩ)	S.D. (kΩ)	Mean (kΩ)	S.D. (kΩ)	
500 µm	R_s (kΩ)	0.540	.053	0.603	.055	0.720
	C_{dl} (µF/cm^2)	3,820	851	57.5	3.23	
	$R_{ct, surf}$ (kΩ)	2.29	.615	585	83.3	
350 µm	R_s (kΩ)	0.710	.159	.921	.185	1.03
	C_{dl} (µF/cm^2)	4,500	909	66.5	4.12	
	$R_{ct, surf}$ (kΩ)	3.93	.877	977	182	
200 µm	R_s (kΩ)	0.880	.211	0.994	.153	1.80
	C_{dl} (µF/cm^2)	6,020	1,031	66.9	4.87	
	$R_{ct, surf}$ (kΩ)	9.15	0.997	1,850	236	
150 µm	R_s (kΩ)	1.20	.346	1.64	.349	2.40
	C_{dl} (µF/cm^2)	7,830	1,440	64.7	5.38	
	$R_{ct, surf}$ (kΩ)	13.1	1.54	2,580	610	
100 µm	R_s (kΩ)	1.72	.641	2.45	.682	3.60
	C_{dl} (µF/cm^2)	8,470	1,970	83.0	8.94	
	$R_{ct, surf}$ (kΩ)	25.4	6.78	4,390	855	
75 µm	R_s (kΩ)	2.41	.950	3.26	1.13	4.80
	C_{dl} (µF/cm^2)	8,520	2,100	102	9.03	
	$R_{ct, surf}$ (kΩ)	49.4	8.49	4,840	970	
50 µm	R_s (kΩ)	4.02	1.23	5.16	1.42	7.20
	C_{dl} (µF/cm^2)	14,300	4,580	145	11.62	
	$R_{ct, surf}$ (kΩ)	89.9	10.2	10,200	1,185	
25 µm	R_s (kΩ)	8.35	1.84	6.06	1.21	14.40
	C_{dl} (µF/cm^2)	27,600	7,590	213	14.72	
	$R_{ct, surf}$ (kΩ)	381	40.9	11,200	1,460	

Table S4. Charge and cathodic charge storage capacity (CSC_c) for $Ti_3C_2T_x$ and Pt microelectrodes.

Contact Diameter	$Ti_3C_2T_x$		Platinum	
	Charge (μC)	CSC (mC/cm^2)	Charge (μC)	CSC (mC/cm^2)
500 μm	40.8 ± 11.4	20.8 ± 5.83	1.31 ± 0.105	0.669 ± 0.054
350 μm	23.9 ± 3.74	24.8 ± 3.89	0.671 ± 0.056	0.698 ± 0.058
200 μm	10.2 ± 2.96	32.5 ± 9.44	0.247 ± 0.053	0.786 ± 0.170
150 μm	7.67 ± 1.68	43.4 ± 9.49	0.164 ± 0.050	0.925 ± 0.281
100 μm	4.69 ± 1.79	59.7 ± 22.8	0.073 ± 0.008	0.936 ± 0.104
75 μm	2.72 ± 1.18	61.5 ± 26.6	0.045 ± 0.005	1.02 ± 0.106
50 μm	1.87 ± 1.06	95.2 ± 53.8	0.037 ± 0.019	1.87 ± 0.955
25 μm	0.588 ± 0.48	119.9 ± 97.3	0.021 ± 0.015	4.29 ± 2.99

Table S5. Capacitances calculated from C_{dl} in the impedance equivalent circuit model and specific capacitance from CV for $Ti_3C_2T_x$ and Pt microelectrodes.

Contact Diameter	$Ti_3C_2T_x$		Platinum	
	Double Layer Capacitance (μF/cm^2)	Specific Capacitance (μF/cm^2)	Double Layer Capacitance (μF/cm^2)	Specific Capacitance (μF/cm^2)
500 μm	3820 ± 851	4060 ± 942	57.5 ± 3.23	49.2 ± 3.85
350 μm	4500 ± 909	4390 ± 892	66.5 ± 4.12	46.0 ± 4.05
200 μm	6020 ± 1031	5550 ± 1180	66.9 ± 4.87	47.5 ± 3.38
150 μm	7830 ± 1440	7590 ± 1240	64.7 ± 5.38	124 ± 7.69
100 μm	8470 ± 1970	8760 ± 1960	83.0 ± 8.94	113 ± 9.92
75 μm	8520 ± 2100	8930 ± 2390	102 ± 9.03	140 ± 13.4
50 μm	14300 ± 4580	13100 ± 4130	145 ± 11.62	381 ± 53.7
25 μm	27600 ± 7590	19300 ± 7640	213 ± 14.72	249 ± 21.1

Table S6. Comparative electrochemical parameters of $Ti_3C_2T_x$, sputtered Pt, gold, PEDOT: PSS / Pt, Sputtered Pt, SIROF, PtIr, and CNT microelectrodes across the microscale from 500 µm to 18 µm.[2–5]

	Diam. (µm)	1 kHz |Z| (kΩ)	10 Hz |Z| (kΩ)	R_s (kΩ)	CSC_c (mC cm^{-2})	$Curr_{max}$ (µA)	CIC_c (µC cm^{-2})	Ref.
$Ti_3C_2T_x$	500	1.97	4.67	0.54	20.8	1880	480	This Work
	150	11.6	28.3	1.20	43.4	231	654	
	100	23.6	58.2	1.72	59.7	97.3	619	
	50	90.8	188	4.02	95.2	30.6	779	
Sputtered Pt	500	1.11	74.4	0.603	0.669	134	34.0	This Work
	150	6.70	529	1.64	0.925	33.6	95.0	
	100	13.7	1,100	2.45	0.936	22.9	146	
	50	49.0	2,670	5.16	1.87	5.60	144	
Gold	500	4.5[a]	300[a]	0.8[a]	0.1[a,b]	300[a,c]	200 [a,b,c]	[2,3]
	150	70[a]	5,000[a]	2.5[a]	0.5[a,b]	30[a,c]	200 [a,b,c]	
	100	170[a]	10,500[a]	4.2[a]	0.8[a,b]	12[a,c]	200 [a,b,c]	
	50	500[a]	30,500[a]	8.5[a]	1[a,b]	3[a,c]	200[a,b,c]	
PEDOT: PSS / Pt	500	2[a]	10.5[a]	1.5[a]	3.5[a,b]	850[a,c]	650[a,b,c]	[2,3]
	150	4.5[a]	190[a]	2.5[a]	5[a,b]	350[a,c]	2700[a,b,c]	
	100	6[a]	300[a]	5.2[a]	5[a,b]	150[a,c]	2700[a,b,c]	
	50	15[a]	1,000[a]	9.5[a]	6[a,b]	40[a,c]	2700[a,b,c]	
Sputtered Pt	500	8[a]	300[a]	1.5[a]	5.1[a,b]	750[a,c]	500 [a,b,c]	[2,3]
	150	75[a]	3,000[a]	2.5[a]	6.0[a,b]	100[a,c]	750 [a,b,c]	
	100	200[a]	5,000[a]	4.2[a]	6.2[a,b]	50[a,c]	850 [a,b,c]	
	50	450[a]	10,500[a]	9.5[a]	8.2[a,b]	10[a,c]	850 [a,b,c]	
SIROF	400	0.501[a]	2.51[a]	N/A	133	5,966[d]	1,900	[4]
PtIr Wire	18	1,100[a]	60,000[a]	N/A	1.2	42.5[d]	150	[5]
CNT Fiber	18	200[a]	4,000[a]	N/A	372	1,570[d]	6,520	[5]

a – Represents values extracted from plots from the respective references

b – Represents a roughly doubled value due to cathodic + anodic normalization

c – Represents values enhanced by positive voltage bias during measurements

d – Represents values calculated from provided pulse-width, CIC, and surface area

Table S7. Max current and cathodic charge injection capacity of $Ti_3C_2T_x$ microelectrodes at varying pulse width.

$Ti_3C_2T_x$ MXene							
**Contact Size**	**Parameter**	**100 µs**	**250 µs**	**500 µs**	**750 µs**	**1000 µs**	**1500 µs**
**500 µm**	**Current (µA)**	3882.2 ± 540.4	2976.8 ± 321.4 µA	1884.1 ± 200.1	1436.8 ± 151.5	1598.8 ± 31.7	
	**CIC (µC/cm^2)**	197.7 ± 27.5	379.0 ± 40.9	479.7 ± 50.9	548.8 ± 57.8	814.2 ± 16.1	
**350 µm**	**Current (µA)**	2504.0 ± 253.3	1638.4 ± 200.0	891.9 ± 128.7	679.2 ± 48.9	1629.4 ± 49.1	
	**CIC (µC/cm^2)**	260.2 ± 26.3	425.7 ± 52.0	463.5 ± 66.9	529.5 ± 38.1	1693.6 ± 51.0	
**200 µm**	**Current (µA)**	673.1 ± 86.2	545.0 ± 91.6	364.9 ± 51.2	313.6 ± 51.5	266.8 ± 36.1	219.2 ± 29.7
	**CIC (µC/cm^2)**	214.2 ± 27.4	433.7 ± 72.9	580.8 ± 81.5	748.7 ± 123.1	849.4 ± 115.0	1046.9 ± 142.2
**150 µm**	**Current (µA)**	497.7 ± 2.05	368.5 ± 70.8	231.1 ± 29.5	196.3 ± 19.2	177.5 ± 14.6	147.5 ± 9.01
	**CIC (µC/cm^2)**	281.6 ± 1.16	521.4 ± 100.1	653.8 ± 83.4	833.5 ± 81.9	1004.5 ± 83.1	1252.1 ± 76.5
**100 µm**	**Current (µA)**	282.2 ± 36.5	180.0 ± 63.3	97.3 ± 15.8	103.2 ± 7.3	94.7 ± 7.1	
	**CIC (µC/cm^2)**	359.3 ± 46.5	573.0 ± 201.6	619.4 ± 100.8	986.0 ± 70.3	1205.9 ± 90.8	
**75 µm**	**Current (µA)**	186.0 ± 31.9	116.3 ± 52.8	59.0 ± 18.8	48.4 ± 11.3	47.3 ± 15.6	
	**CIC (µC/cm^2)**	421.08 ± 72.3	658.1 ± 299.1	668.1 ± 213.3	823.2 ± 192.1	1072.8 ± 355.3	
**50 µm**	**Current (µA)**	60.3 ± 10.7	45.9 ± 9.003311	30.6 ± 4.18	24.9 ± 5.3	21.5 ± 2.9	
	**CIC (µC/cm^2)**	307.2 ± 54.9	584.6 ± 114.6	779.2 ± 106.5	952.4 ± 204.6	1096.3± 150.0	
**25 µm**	**Current (µA)**	11.9 ± 4.1	18.5 ± 12.1	4.88 ± 1.9	4.2 ± 2.38	5.7 ± 2.8	
	**CIC (µC/cm^2)**	243.8 ± 83.6	943.4 ± 616.3	497.0 ± 195.5	650.2 ± 364.5	1172.9 ± 574.2	

Table S8. Max current and cathodic charge injection capacity of sputtered Pt microelectrodes at varying pulse width.

Sputtered Platinum							
Contact Size	Parameter	100 µs	250 µs	500 µs	750 µs	1000 µs	1500 µs
500 µm	Current (µA)	623.7 ± 69.7	364.3 ± 51.7	133.7 ± 8.35	426.8 ± 19.2	726.7 ± 44.5	
	CIC (µC/cm²)	31.7 ± 3.5	46.3 ± 6.5	34.0 ± 2.12	163.0 ± 7.3	388.7 ± 79.1	
350 µm	Current (µA)	472.9 ± 24.8	440.6 ± 118.1	101.6 ± 3.0	397.9 ± 1.88	626.8 ± 44.4	
	CIC (µC/cm²)	49.1 ± 2.58	114.4 ± 30.7	52.8 ± 1.58	310.2 ± 1.47	634.2 ± 73.7	
200 µm	Current (µA)	122.0 ± 19.5	142.1 ± 51.3	67.6 ± 15.9	74.5 ± 2.59	76.5 ± 2.91	75.6 ± 2.83
	CIC (µC/cm²)	38.8 ± 6.21	113.1 ± 40.9	107.6 ± 25.3	178.0 ± 6.20	243.6 ± 9.29	361.1 ± 13.5
150 µm	Current (µA)	89.2 ± 14.1	102.1 ± 26.7	33.6 ± 2.84	74.4 ± 2.75	74.3 ± 2.52	76.3 ± 3.46
	CIC (µC/cm²)	50.5 ± 7.98	144.4 ± 37.9	95.0 ± 8.04	315.9 ± 11.7	420.6 ± 14.1	648.1 ± 11.0
100 µm	Current (µA)	37.6 ± 8.8	27.488 ± 13.7	22.9 ± 8.06	21.35 ± 8.09	18.0 ± 2.64	
	CIC (µC/cm²)	47.9 ± 11.2	87.3 ± 43.6	146.0 ± 51.3	203.8 ± 77.3	229.8 ± 33.7	
75 µm	Current (µA)	29.1 ± 11.6	23.6 ± 9.4	15.0 ± 4.21	14.3 ± 0.28	18.2 ± 4.3	
	CIC (µC/cm²)	65.9 ± 26.3	133.6 ± 53.4	170.2 ± 47.7	243.32 ± 4.8	413.0 ± 97.5	
50 µm	Current (µA)	29.1 ± 19.6	8.3 ± 4.8	5.6 ± 2.9	4.6 ± 2.4	4.3 ± 1.38	
	CIC (µC/cm²)	148.4 ± 100.2	106.1 ± 61.3	143.9 ± 74.4	176.9 ± 95.2	221.5 ± 70.4	
25 µm	Current (µA)	4.83 ± 0.93	2.76 ± 1.03	1.38 ± 0.55	1.64 ± 0.61	3.11 ± 2.09	
	CIC (µC/cm²)	98.1 ± 19.0	140.7 ± 52.6	140.8 ± 56.5	250.6 ± 93.4	635.0 ± 209.1	

Table S9. Tentative estimates of minimum electrode size based on representative recording and stimulation requirements. Model equations arising from impedance fitting and CIC values with representative stimulation amplitudes set as magnitude guidelines from other bodies of work.[6–10] Equations from this table are derived from the impedance fit calculation from Figure 2, as well as from the calculation of CIC in Figure 5. Both equations and their corresponding variables are provided below in supplementary equations.

	Recording $D = \left(\frac{\lvert Z\rvert}{e^b}\right)^{1/m}$		Stimulation $D = 2\sqrt{\frac{A_{\min}\,pw}{\pi\,CIC}}$		
**Condition**	Local Fields ($\lvert Z_{10\ Hz}\rvert$ > 1 MΩ)	Multi-Unit Activity ($\lvert Z_{1\ kHz}\rvert$ > 1 MΩ)	Mouse Model A_{min} = 5 µA	Swine Model A_{min} = 50 µA	Human Model A_{min} = 100 µA
**MXene**	*m= -1.57, b= 18.19* **D = 16 µm**	*m= -1.65, b= 17.73* **D = 11 µm**	*pw=500, CIC=592* **D = 23 µm**	*pw=500, CIC=592* **D = 73 µm**	*pw=500, CIC=592* **D = 104 µm**
**Platinum**	*m= -1.39, b= 19.93* **D = 81 µm**	*m= -1.57, b= 16.69* **D = 6 µm**	*pw= 500, CIC=111* **D = 54 µm**	*pw=500, CIC=111* **D = 169 µm**	*pw=500, CIC=111* **D = 240 µm**

Table S10. Influence of processing parameters on the performance of $Ti_3C_2T_x$ MXene microelectrodes.[11–15]

Contact Scale	MXene Prep	Diameter (µm)	Area (mm^2)	Impedance $\lvert Z_{10Hz}\rvert$ (kΩ)	Area-normalized $\lvert Z_{10Hz}\rvert$ (Ω mm^2)	Ref.
MICRO SCALE	Thick spray-coat	75	0.00442	117	516.9	This Work
	Thin spray-coat	50x50	0.00250	5,000[a]	12,500.0	[11]
	Spin-coat	50x50	0.00250	500[a]	1250.0	[12]
	Dip-coat	100	0.00785	100[a]	785.4	[13]
MACRO SCALE	Thick spray-coat	3,000	7.06858	0.350[a]	2,474.2	[14]
	Thin spray-coat	3,000	7.06858	0.750[a]	5,301.8	[14]
	Blade-coat	3,000	7.06858	0.300[a]	2,120.7	[14]
	MXtrode	3,000	7.06858	0.200[a]	1,413.8	[15]

a – Represents values extracted from plots from the respective references

SUPPLEMENTARY EQUATIONS

Equation 1: Diameter scaling with impedance. Where |Z| is the impedance limit of interest, m, and b are derived from the impedance fit, calculated in Figure 2.

$$D = \left(\frac{|Z|}{e^{b}}\right)^{1/m}$$

Equation 2: Diameter scaling with charge injection capacity. Where A_{min} is the minimum current amplitude of interest, *pw* is the pulse-width of the cathodic current pulse, and *CIC* is the charge injection capacity for that material, calculated and provided in Figure 5 for both MXene and Platinum.

$$D = 2\sqrt{\frac{A_{\min} \cdot pw}{\pi\, CIC}}$$

REFERENCES

1. Suesserman, M. F., Spelman, F. A. & Rubinstein, J. T. In vitro measurement and characterization of current density profiles produced by nonrecessed, simple recessed, and radially varying recessed stimulating electrodes. *IEEE Transactions on Biomedical Engineering* **38**, 401–408 (1991).
2. Ganji, M., Tanaka, A., Gilja, V., Halgren, E. & Dayeh, S. A. Scaling Effects on the Electrochemical Stimulation Performance of Au, Pt, and PEDOT:PSS Electrocorticography Arrays. *Adv. Funct. Mater.* (2017).
3. Ganji, M. *et al.* Scaling Effects on the Electrochemical Performance of poly(3,4-ethylenedioxythiophene (PEDOT), Au, and Pt for Electrocorticography Recording. *Adv. Funct. Mater.* (2017).
4. Cogan, S. F. *et al.* Sputtered iridium oxide films for neural stimulation electrodes. *J Biomed Mater Res* **89B**, 353–361 (2009).
5. Vitale, F., Summerson, S. R., Aazhang, B., Kemere, C. & Pasquali, M. Neural Stimulation and Recording with Bidirectional, Soft Carbon Nanotube Fiber Microelectrodes. *ACS Nano* **9**, 4465–4474 (2015).
6. Lycke, R. *et al.* Low-threshold, high-resolution, chronically stable intracortical microstimulation by ultraflexible electrodes. *Cell Rep* **42**, 112554 (2023).
7. Cogan, S. F., Ludwig, K. A., Welle, C. G. & Takmakov, P. Tissue damage thresholds during therapeutic electrical stimulation. *J Neural Eng* **13**, 021001 (2016).
8. Urdaneta, M. E. *et al.* The Long-Term Stability of Intracortical Microstimulation and the Foreign Body Response Are Layer Dependent. *Front. Neurosci.* **16**, (2022).
9. Kim, W. J. *et al.* Superior cervical ganglion stimulation results in potent cerebral vasoconstriction in swine. *J Vasc Interv Neurol* **13**, 35–41 (2022).
10. Hughes, C. L. *et al.* Perception of microstimulation frequency in human somatosensory cortex. *eLife* **10**, e65128 (2021).
11. Driscoll, N. *et al.* Two-Dimensional Ti3C2 MXene for High-Resolution Neural Interfaces. *ACS Nano* (2018).
12. Shankar, S. *et al.* Effect of the deposition process on the stability of $Ti_3 C_2 T_x$ MXene films for bioelectronics. *2D Mater.* **10**, 044001 (2023).
13. Shankar, S. *et al.* Transparent MXene Microelectrode Arrays for Multimodal Mapping of Neural Dynamics. *Adv Healthcare Materials* **14**, 2402576 (2025).
14. Bi, L. *et al.* Soft, Multifunctional MXene-Coated Fiber Microelectrodes for Biointerfacing.
15. Driscoll, N. *et al.* MXene-infused bioelectronic interfaces for multiscale electrophysiology and stimulation. *Sci. Transl. Med.* **13**, eabf8629 (2021).
